# Exosomal miR‐21‐5p derived from bone marrow mesenchymal stem cells promote osteosarcoma cell proliferation and invasion by targeting PIK3R1

**DOI:** 10.1111/jcmm.17024

**Published:** 2021-11-05

**Authors:** Jin Qi, Ruihao Zhang, Yapeng Wang

**Affiliations:** ^1^ Department of Orthopaedics Lanzhou University Second Hospital Lanzhou People’s Republic of China; ^2^ Orthopaedics Key Laboratory of Gansu Province Lanzhou People’s Republic of China

**Keywords:** exosomes, mesenchymal stem cells, miR‐21‐5p, osteosarcoma, PI3K/Akt/mTOR pathway, PIK3R1

## Abstract

Mesenchymal stem cells (MSCs) are a class of pluripotent cells that can release a large number of exosomes which act as paracrine mediators in tumour‐associated microenvironment. However, the role of MSC‐derived exosomes in pathogenesis and progression of cancer cells especially osteosarcoma has not been thoroughly clarified until now. In this study, we established a co‐culture model for human bone marrow‐derived MSCs with osteosarcoma cells, then extraction of exosomes from induced MSCs and study the role of MSC‐derived exosomes in the progression of osteosarcoma cell. The aim of this study was to address potential cell biological effects between MSCs and osteosarcoma cells. The results showed that MSC‐derived exosomes can significantly promote osteosarcoma cells’ proliferation and invasion. We also found that miR‐21‐5p was significantly over‐expressed in MSCs and MSC‐derived exosomes by quantitative real‐time polymerase chain reaction (qRT‐PCR), compared with human foetal osteoblastic cells hFOB1.19. MSC‐derived exosomes transfected with miR‐21‐5p could significantly enhance the proliferation and invasion of osteosarcoma cells in vitro and in vivo. Bioinformatics analysis and dual‐luciferase reporter gene assays validated the targeted relationship between exosomal miR‐21‐5p and PIK3R1; we further demonstrated that miR‐21‐5p‐abundant exosomes derived human bone marrow MSCs could activate PI3K/Akt/mTOR pathway by suppressing PIK3R1 expression in osteosarcoma cells. In summary, our study provides new insights into the interaction between human bone marrow MSCs and osteosarcoma cells in tumour‐associated microenvironment.

## 1 INTRODUCTION

Osteosarcoma (OS) is one of the most common primary malignant and aggressive type of bone cancer in adolescents. Recurrence and metastasis are the main reasons for the unsatisfactory treatment of OS.^1^ However, the mechanism of proliferation and invasion of OS is still not clear. As we all know, the biological behaviour of cancer is not only determined by cancer cells alone, and stromal cell in tumour‐associated microenvironment acts on cancer cells to promote cancer proliferation and invasion, which has become more and more of a concern. As a non‐tumour cell, mesenchymal stem cells (MSCs) may participate in processes of OS proliferation and invasion in OS‐associated microenvironment.^2^ MSCs are a kind of non‐hematopoietic progenitor cells with multipotent and self‐renewable abilities which reside in bone marrow, umbilical cord, placenta tissue, adipose tissue, etc.^3^, ^4^, ^5^, ^6^ Furthermore, MSCs have biological characteristics of migrating to the cancer site where they participate in the formation of tumour‐associated microenvironment and have always been studied in the field of cancer pathogenesis and cancer therapy.^7^, ^8^ MSCs not only provide the microenvironment for cancer cells, but also enhance cancer progression and invasion.^9^ It has been found that the aneuploidy and genomic loss can transform MSCs into OS cells, indicating that OS cells may originate from bone marrow MSCs.^10^ Studying the role of MSCs in OS proliferation and invasion is of great significance to elucidate the mechanism of OS progression.

Classically, MSCs play its role mainly through cell contact‐dependent mechanisms and soluble factors.^11^ However, further studies have found that MSCs could secrete a large number of exosomes through paracrine mechanism, which can change the microenvironment around target cells, thus regulating the biological functions of cell proliferation and differentiation.^12^, ^13^, ^14^ Exosomes are small, lipid bilayer membrane vesicles with a diameter of about 30–100 nm and contain a lot of specific biologically active molecules such as nucleic acids, lipid, and proteins.^15^ Moreover, exosomes carry a variety of types of RNA, DNA, protein and other signal molecules of donor cells.^16^ Its main biological function is to transmit the signal molecules to recipient cells, thus forming the information exchange and transmission between cells.^17^ In addition, exosomes are involved in the pathological processes of various diseases, including cancer.^18^ For example, studies have shown exosomes from different cell types involved in cancer cells proliferation, angiogenesis, differentiation and metastasis.^19^, ^20^, ^21^, ^22^

In previous studies, we found that MSC‐derived exosomes enhance the proliferation of OS,^23^ but we have not yet elucidated the specific molecular biological mechanism. Recently, it has been found that the expression of miR‐21‐5p increased during the osteogenic differentiation of MSCs.^24^ MiR‐21‐5p is considered to be an oncogene that promotes tumour proliferation and invasion.^25^ Here, we found that MSC‐derived exosomes may be rich in miR‐21‐5p, which further affects the biological characteristics of OS, and the exosomes protect miR‐21‐5p from RNase degradation in OS‐associated microenvironment. Bioinformatics prediction found that PIK3R1 is the target gene of miR‐21‐5p and is also responsible for encoding an important regulatory subunit (p85α) of phosphatidylinositol 3‐kinases(PI3K), PI3K forms a heterodimeric protein complex composed of p85 regulatory subunit and catalytic p110 subunit encoded by the PIK3CA.^26^, ^27^ Furthermore, PIK3CA oncogene carrying hotspot mutation has carcinogenic activity. In contrast, PIK3R1 seems to play a tumour‐suppressor role because PI3K subunit p85α adjusts and stabilizes p110.^27^, ^28^, ^29^ P85 type also has other two subunits: PIK3R2 and PIK3R3. Classically, p85α and p85β are considered to be similar proteins associated with activated receptor tyrosine kinases (RTK) that induce PI3K activation; however, PIK3R1/p85α is the most abundant subtype in tumour‐free tissues, but its expression is reduced in cancer, which has a tumour‐suppressor function.^30^ In contrast, PIK3R2/p85β expression is upregulated in cancer which is regarded as a tumour driver.^31^, ^32^, ^33^ PI3K activation is induced by p85 binding to activated receptor tyrosine kinase (RTK).^34^ PI3K/Akt/mTOR is the signalling pathways closely related to apoptosis and cell proliferation, which is usually highly activated in OS.^35^ In this study, we hypothesized that miR‐21‐5p‐abundant MSC‐derived exosomes might participate in OS cells’ proliferation and invasion through activated PI3K/Akt/mTOR signalling pathway, and miR‐21‐5p expression in MSC‐derived exosomes may serve as a therapeutic target for OS in the future.

## 2 MATERIALS AND METHODS

### 2.1 Cell culture

Human U2OS, MG63 and Human osteoblasts (hFOB1.19) (purchased from Cell Bank, Chi Scientific, Inc.) were cultured in DMEM with 10% foetal bovine serum and 1% penicillin‐streptomycin. All cells were cultured in an incubator with a humidity atmosphere of 5% CO_2_ at 37℃. Following the guidelines of the International Conference on Harmonization (ICH) E‐6 Good Clinical Practice and approved by the ethics committee of Lanzhou university second hospital (#2018A‐018, Lanzhou, China) and informed written consent, primary human MSCs were isolated from bone marrow of 6 patients (15–37 years old) who have been diagnosed as OS and were adhere‐wall cultured to isolation, and it has been described in detail in our previous study.^23^ MSCs were cultured in complete DMEM/F12 supplemented with 10% exosome‐depleted foetal bovine serum. When the cultured MSCs reached 80%–90% confluence, culture supernatants were harvested. The third passage of MSCs was collected for in vitro phenotype analysis.

### 2.2 Identification of MSCS‐ and MSC‐derived exosomes

Cellular surface antigens of MSCs were examined with flow cytometry, and it has been described in detail in previous experiment.^36^ The osteogenic and adipogenic differentiation of MSCs was further performed by using the differentiation media and detected by Oil red O staining and Alizarin red staining (Cyagen Bioscience, Inc.). Cellular surface antigens were examined by using a flow cytometry of MSCs for CD19, CD29, CD90, CD44, CD73, CD105 and CD133 markers. MSC‐derived exosomes were isolated according to the exosome extraction protocol.^37^ In short, cell culture supernatants at 300 × *g* centrifugation for 10 min to remove cells, to 2000 × *g* centrifuged for 10 min to remove dead cells, and at 10,000 × *g* for 30 min to remove cell debris and retain the supernatant for further ultracentrifugation. At twice 100,000 × *g* ultracentrifugation for 70 min, the pellets (exosomes) were retained, and the supernatant was discarded. Purified exosomes were negatively stained with uranyl acetate by means of floating method and observed by transmission electron microscope. The particle size distribution of exosomes was characterized and quantified by NanoSight LM10 system (NanoSight).

### 2.3 Co‐culture experiments

MSCs were seeded in a 6‐well plate of a density of 1 × 10^4^ cells/cm^2^, and OS cells seeded in 6‐well chambers with 0.4 μm polycarbonate membrane pores. The membrane was permeable, and cells could not pass through the membrane, but the cytokines secreted by cells could pass through. Cells were co‐cultured for 2 weeks, and then, induced MSCs were collected for further experiments.

### 2.4 Choice of differentially expressed mirnas list using heat map analysis

We obtained the microarray data from Gene Expression Omnibus (GEO, http://www.ncbi.nlm.nih.gov/geo/), and the GEO accession Nos. are GSE58027 and GSE89930. The heat map of miRNAs showed significant differences using the hierarchical clustering method.^38^

### 2.5 Data mining in oncomine database

Oncomine database (https://www.oncomine.org/resource/login.html) is a publicly accessible online cancer microarray database, which helps to promote the research of genome‐wide expression analysis.^39^ We used Oncomine database to determine the transcription level of PIK3R1 gene in sarcoma by searching the expression level of PIK3R1 mRNA (log2 transformation) in sarcoma and normal tissues (tumour‐free tissues).

### 2.6 PKH26‐labelled exosome and confocal microscopy

PKH26 was used to label exosomes, and 4′,6‐diamidino‐2‐phenylindole (DAPI) was used to label OS cell nuclei. The process of OS cells uptake of exosomes was observed under a Nikon Eclipse 80i confocal fluorescence microscope.

### 2.7 Treatment of MSCs with GW4896

For the inhibition of exosome generation, MSCs were pre‐treated with exosome‐free media containing 10 μM GW4869 (Umibio) for 24–48 h. When wall‐adhered MSCs reached 90% confluence, the culture supernatants were collected for further cell proliferation assays.

### 2.8 Cell proliferation assays

After reaching 80% confluence, OS cells inoculate into 96‐well plates (100 μl/well). OS cells were treated with or without 40 μg of MSC‐derived exosomes. In order to eliminate the effect of MSC‐derived exosomes on OS cells, GW4869‐treated MSCs were added to OS cells as MSC‐GW4869 group. Cell growth was measured with a 10 μl cell counting kit (ZP328; ZOMANBIO) according to the manufacturer's instruction (CCK‐8 assay). Absorbance was read at 450 nm using a microplate reader (Tecan Infinite 200 Pro). OS cells were co‐cultured with different culture medium; then, OS cells labelled with CFSE (Thermo Fisher Scientific, Inc.) and detected by flow cytometry (Becton Dickinson) according to the manufacturer's instruction.

### 2.9 Wound‐healing assay

OS cells were divided into a 6‐well plate at a rate of 1 × 10^5^ cells per well. When cells grew to 95% confluence, monolayers were wounded by a sterile 10 μl plastic micropipette tip, washed and added different culture medium according to the needs of the experiment. The width of the scratch gap under the microscope was observed and photographed.

### 2.10 Transwell invasion assay

OS cell invasion experiments were conducted using 24‐well chambers with 8.0‐μm PET membrane pores. The number of cells invading through the membrane was calculated in 10 regions/well.^40^

### 2.11 Luciferase reporter assays

OS cells were transfected with miR‐21‐5p mimics/inhibitor or different combinations of control sequence, psiCHECK‐2‐PIK3R1 3’UTR‐WT and psiCHECK‐2‐PIK3R1 3’UTR‐Mut for 48 h. The dual‐luciferase reporter kit (Promega) was to evaluate relative luciferase activity.^41^

### 2.12 RNA extraction and real‐time PCR analysis

RNA extraction and real‐time quantitative real‐time polymerase chain reaction (PCR) analysis were performed as mentioned earlier.^42^ For microRNA quantification, total RNA was reverse‐transcribed by TaqMan MicroRNA Reverse Transcription Kit (Applied Biosystems), and then, TaqMan miRNA assay was performed according to the manufacturer's agreement (Applied Biosystems). Quantification of miR‐21‐5p was performed with a stem‐loop real‐time PCR miRNA kit (Ribobio). U6 was used as the internal reference for qRT‐PCR.

### 2.13 Western blotting

Protein extraction and Western blotting were performed as described previously.^43^ The following antibodies were used according to the manufacturer's instructions: Calreticulin, CD63 (Abcam, Inc.). Proteins were detected using specific antibodies (Abcam, Inc.). The expressions of Bcl‐2, Bax, PIK3R1, PIK3R2 and PI3K/AKT/mTOR pathway‐related proteins in human OS cell lines were also assessed by WB assays. The primary antibodies including Bcl‐2, Bax, PIK3R1, PIK3R2, phosphorylated (p)‐PI3K, PI3K, p‐Akt, Akt, p‐mTOR, Mtor and β‐actin were used as a loading control.

### 2.14 In vivo tumour growth analysis

All experiments were approved by the Animal Research Ethics Committee of Lanzhou university second hospital. In the assessment of the effect of MSC‐derived exosomes on OS growth in vivo, all nude mice were inoculated subcutaneously with MG63 cells (5 × 10^6^) through subcutaneous injection. After 1 week of tumour growth, mice were randomly divided into the following groups (*n* = 6): OS cells mixed with miR‐21‐5p‐exosomes (MSCs were treated with miR‐21‐5p mimic to purify miR‐21‐5p‐exosomes) and OS cells mixed with miR‐NC‐exosomes. In control mice, we injected intra‐tumour, the same volume of PBS (vehicle) alone. Each mice (*n *= 6/group) was injected with MSC‐derived exosomes suspension (400 μg/ml) by intra‐tumour injection every week. After injection, calliper was used to measure the tumour size, and the following formula was used to calculate the tumour volume: Tumour volume (mm^3^) = 0.52 × width (mm)^2^ × length (mm). At the end of the 5th week, nude mice were sacrificed using 20% overdose pentobarbital.

### 2.15 Statistical analyses

The data were presented as mean ± standard deviation and statistically analysed by IBM SPSS Statistics 21.0 software (URL: https://www.ibm.com/support/pages/node/723919) and GraphPad Prism 5.0 software (URL: https://download.csdn.net/download/qq_31202341/9095763). The heat maps were made by heml 1.0.3.7 software (URL: https://download.pchome.net/development/html/download‐13816.html). *p*‐values <0.05 were considered to be statistically significant. All experiments were performed using at least three independent experiments.

## 3 RESULT

### 3.1 Characterization of MSCs‐ and MSC‐derived exosomes

**FIGURE 1:**
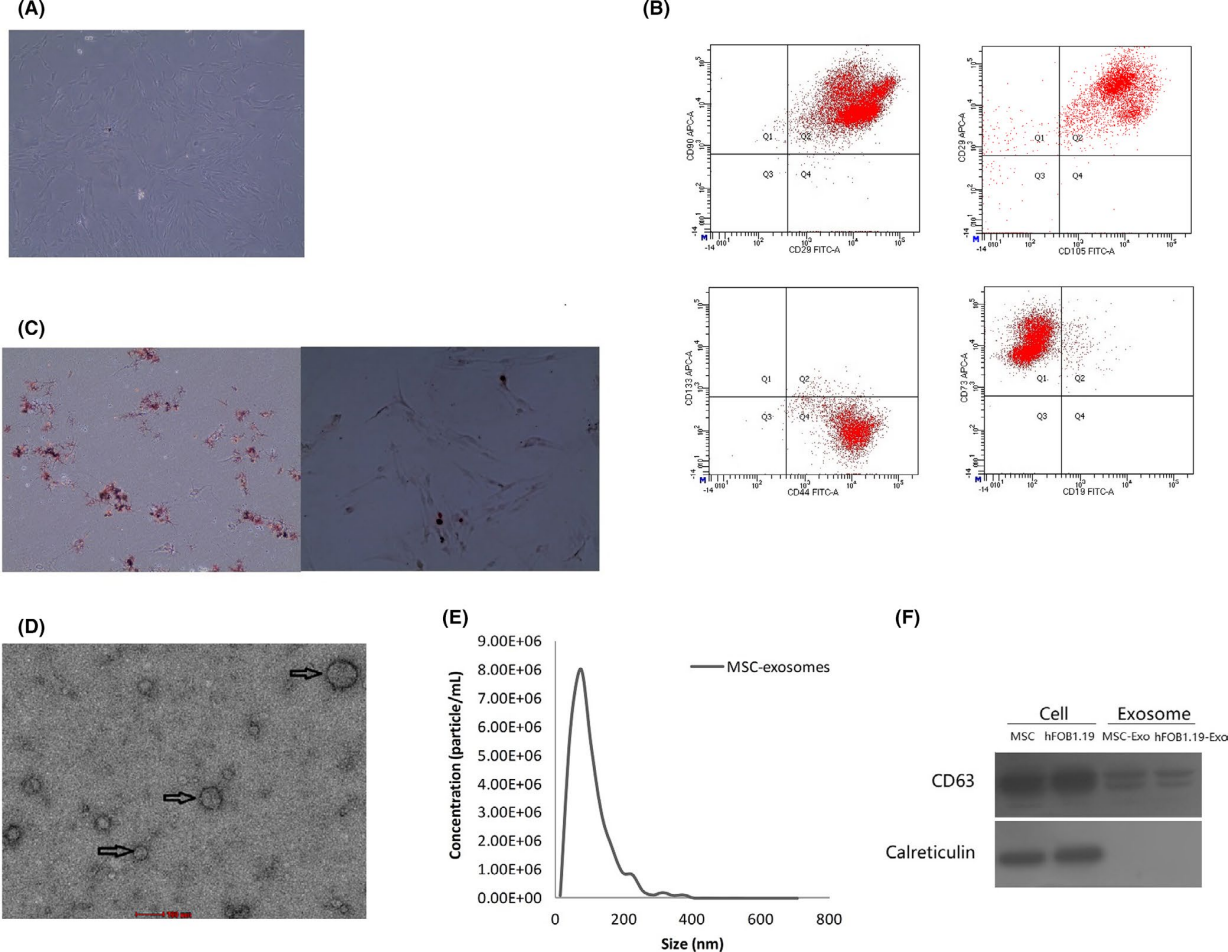
Characterizations of human bone marrow MSCs and MSC‐derived exosomes. (A) Under an optical microscope, human bone marrow MSCs grow in a fibroblast‐like, spindle‐shaped morphology. (B) Flow cytometry to detect the phenotype of human bone marrow MSCs (Q1: APC+/FITC−, Q2: APC+/ FITC+, Q3: APC−/ FITC−, Q4: APC−/ FITC+). (C) Oil red O staining showed that a small amount of lipid droplets in MSCs display bright red under microscope, and a large number of orange‐red calcium deposits were observed after alizarin red S staining. (D) Transmission electron microscopy showed that MSC‐derived exosomes were typically small and round nanoparticles with a diameter of 40–80 nm. The scale was 100 nm. (E) Size distributions of MSC‐derived exosomes were identified using NanoSight analysis. (F) Exosomal positive marker CD63 was detected in MSC‐ and hFOB1.19‐exosomes, whereas negative marker calreticulin was not detected

MSCs from bone marrow of patients diagnosed with OS began to adhere to the wall after inoculation 24 h, and after 2–3 passages, MSCs displayed fibroblast‐like cell adherent growth and grew in a long spindle shape (Figure 1A). Flow cytometry showed that MSCs expressed CD29, CD90, CD44, CD73 and CD105 markers, but negative results for CD19 and CD133 (Figure 1B). After 2 weeks of osteogenic and lipogenic induction, MSCs differentiated into osteocytes and adipocytes, stained with alizarin red S and oil red (Figure 1C). Transmission electron microscopy showed that a large number of MSC‐derived exosomes were typical small spherical nanoparticles with a diameter of 40‐80nm (Figure 1D). NanoSight analysis showed the diameter distribution of exosomes ranged from 30 to 165 nm, and exosomes with diameter of 75 nm had a peak (Figure 1E). MSC‐derived exosomes express primarily surface marker CD63, and Calreticulin, an intracellular protein, was negatively expressed (Figure 1F). Calreticulin is a highly conserved chaperone protein of the endoplasmic reticulum that has specificity towards glycoprotein substrates, which exists in all cells except red blood cells in high‐grade biology.^44^

### 3.2 Characterization of exosome internalization by OS cells

**FIGURE 2:**
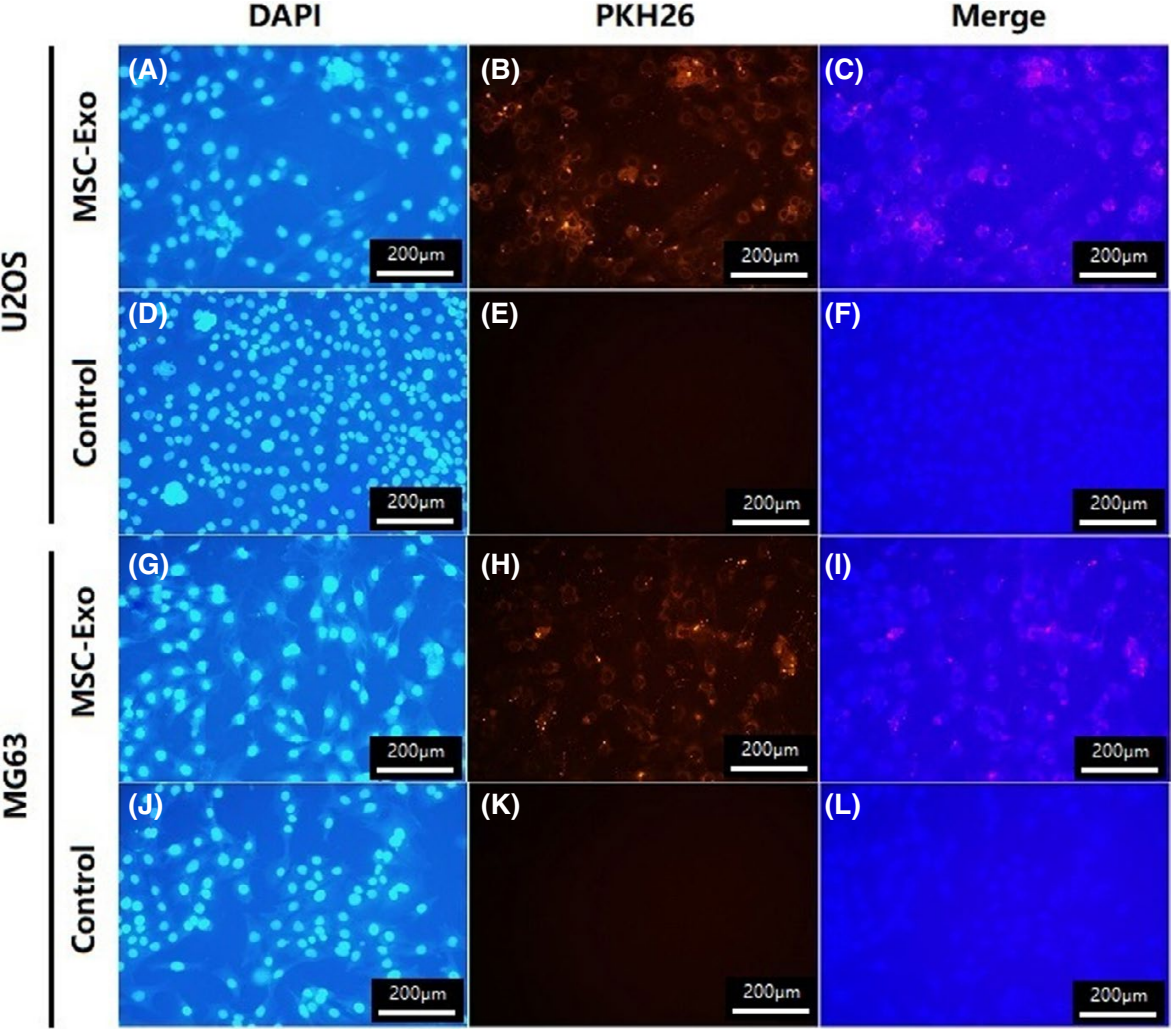
MSC‐derived exosomes internalization by U2OS and MG63. At room temperature, MSC‐derived exosomes were labelled with PKH26 (B, H), and the supernatant without exosomes was also labelled with PKH26 as a negative control (E, K). U2OS and MG63 were stained with DAPI (A, D, G, J). U2OS and MG63 were imaged by confocal fluorescence microscopy (C, I)

To investigate whether MSC‐derived exosomes were internalized by OS cells, we used the fluorescent dye, PKH26, to labelled MSC‐derived exosomes, and OS cell nuclei were stained by DAPI. Under the confocal laser microscope, most of OS cells could be observed in a red fluorescence signal, the fluorescent exosomes are mainly located in the cytoplasm ofU2OS and MG63. In order to eliminate PKH26 dye contamination in U2OS and MG63, exosome‐free supernatant was also stained by PKH26 and no red fluorescence was detected (Figure 2).

### 3.3 MSC‐derived exosomes promote OS cells proliferation and invasion

**FIGURE 3:**
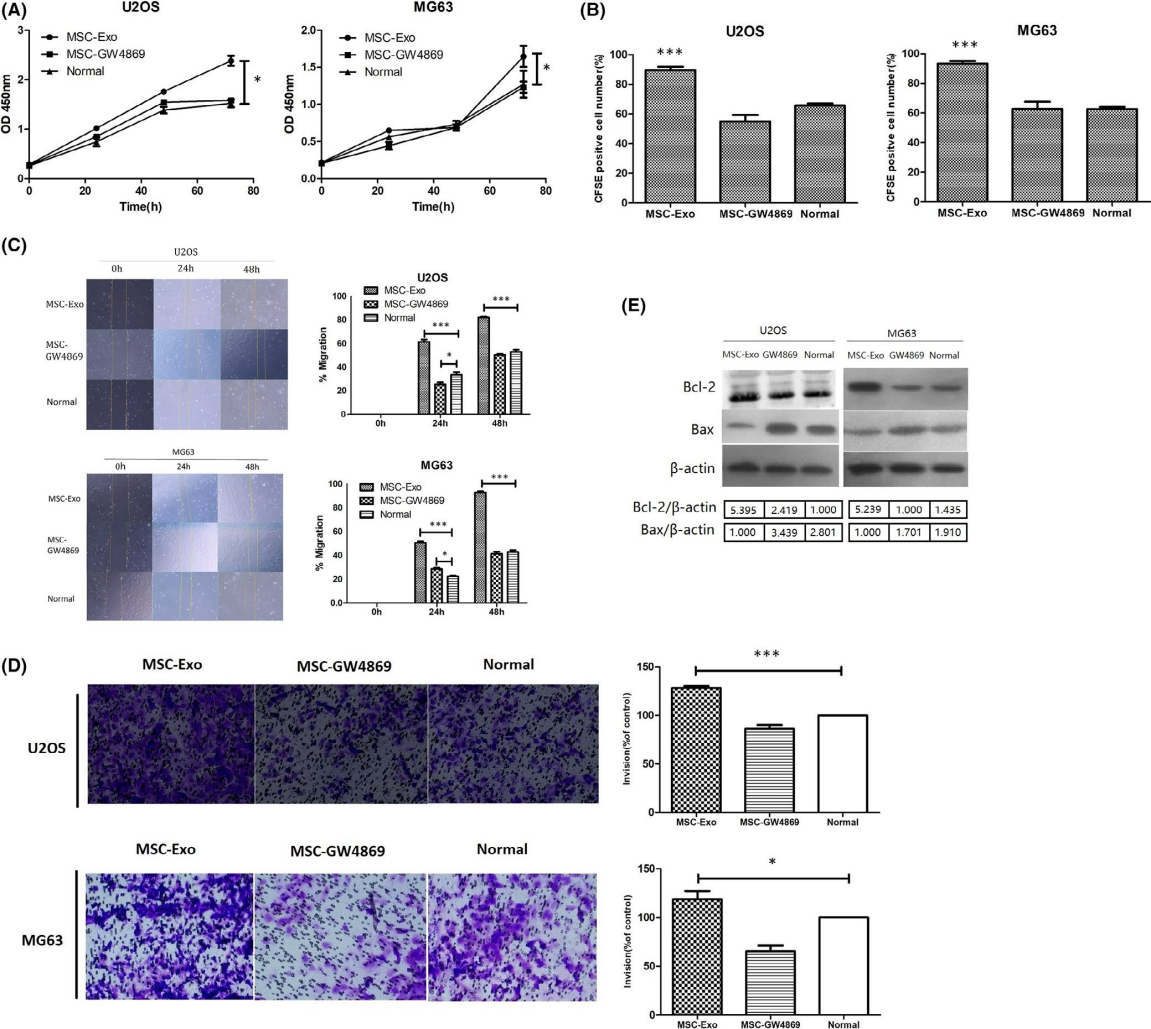
MSC‐derived exosomes increase OS cells proliferation and invasion in vitro. (A) U2OS and MG63 were co‐cultured with MSC‐derived exosomes and MSC‐GW4869 for 24, 48 and 72 h and then subjected to CCK‐8 analyses. Compare with normal group (untreated group), the proliferation rates of both U2OS and MG63 cells were higher after MSC‐derived exosomes treatment at 72 h (*p *< 0.05), whereas there was no significant difference between MSC‐GW4869 group and normal group (*p *> 0.05). (B) From CFSE fluorescence labelling system, the proportions of U2OS and MG63 proliferation in MSC‐Exo groups were significantly higher than that of normal groups and MSC‐GW4869 groups (*p *< 0.001). (C) U2OS and MG63 cells migration after exposure to MSC‐derived exosomes compared with MSC‐GW4869‐treated and MSC‐GW4869‐untreated controls, examined by scratch‐healing assay. The wound‐healing assay showed that the migration ability of U2OS and MG63 in MSC‐derived exosomes groups was greater at 24 and 48 h (*p *< 0.001). (D) After 24 h, the number of U2OS and MG63 that migrated to the sub‐chamber of the 8 μm pore membrane by counting the number of cells in each field was analysed. Compared with MSC‐GW4869 groups and normal groups, the number of U2OS and MG63 was significantly increased 1.283‐fold and 1.187‐fold, respectively (*p *< 0.05). *p < 0.05, ***p < 0.001. (E) The protein levels of Bcl‐2 and Bax in U2OS and MG63 cells of various groups, which were evaluated by Western blot

In order to research the effect of MSC‐derived exosomes on the proliferation of OS cells (U2OS, MG63) in vitro, OS Cells were measured at 24, 48 and 72 h after MSC‐derived exosomes and MSC‐GW4869 (blockage of exosome generation) treatment. The CCK‐8 assay showed that compared with normal group (untreated group), the proliferation rates of both U2OS and MG63 cells were highly observed after MSC‐derived exosomes treatment for 72 h (*p *< 0.05, Figure 3A). Therefore, it was suggested that MSC‐derived exosomes promoted OS cells proliferation. These results were further validated by CFSE fluorescence labelling system. After 72 h of co‐culture, the proliferation of U2OS and MG63 was evaluated by CFSE fluorescence labelling system. The results showed that the proportions of U2OS and MG63 proliferation in normal group were separately 65.72 ± 1.33% and 62.6 ± 1.41%, in MSC‐Exo group were 89.64 ± 2.14% and 93.40 ± 1.75%, and in MSC‐GW4869 group were 54.95 ± 4.39% and 62.8 ± 4.75%. There were statistical differences between MSC‐Exo group and normal group (*p *< 0.05), while there was no difference between MSC‐GW4869 group and normal group (*p *> 0.05, Figure 3B). Scratch wound‐healing assay was conducted by evaluating the effect of MSC‐derived exosomes on the invasive abilities of U2OS and MG63. Although no obvious differences were observed between MSC‐GW4869 groups and normal groups at 48 h, the rate of wound healing in U2OS and MG63 was significantly accelerated in the MSC‐Exo groups at 24 and 48 h (*p *< 0.001, Figure 3C). Transwell analysis further verified these results. Compared with MSC‐GW4869 group and the normal group, the number of transmigrated U2OS and MG63 was significantly increased to 1.283‐fold and 1.187‐fold, respectively, after co‐culture with MSC‐derived exosomes for 24 h (*p *< 0.05, Figure 3D). To further investigate the protein changes in proliferation and apoptosis of U2OS and MG63, Western blot analysis found that compared with MSC‐GW4869 and normal group, MSC‐derived exosomes can upregulate the expression of Bcl‐2 in U2OS and MG63, while the expression of Bax was downregulated (Figure 3E). These results indicated that MSC‐derived exosomes can effectively promote OS cells invasion and proliferation.

### 3.4 Bioinformatics prediction of exosomal MIR‐21‐5p and targeting gene PIK3R1

**FIGURE 4:**
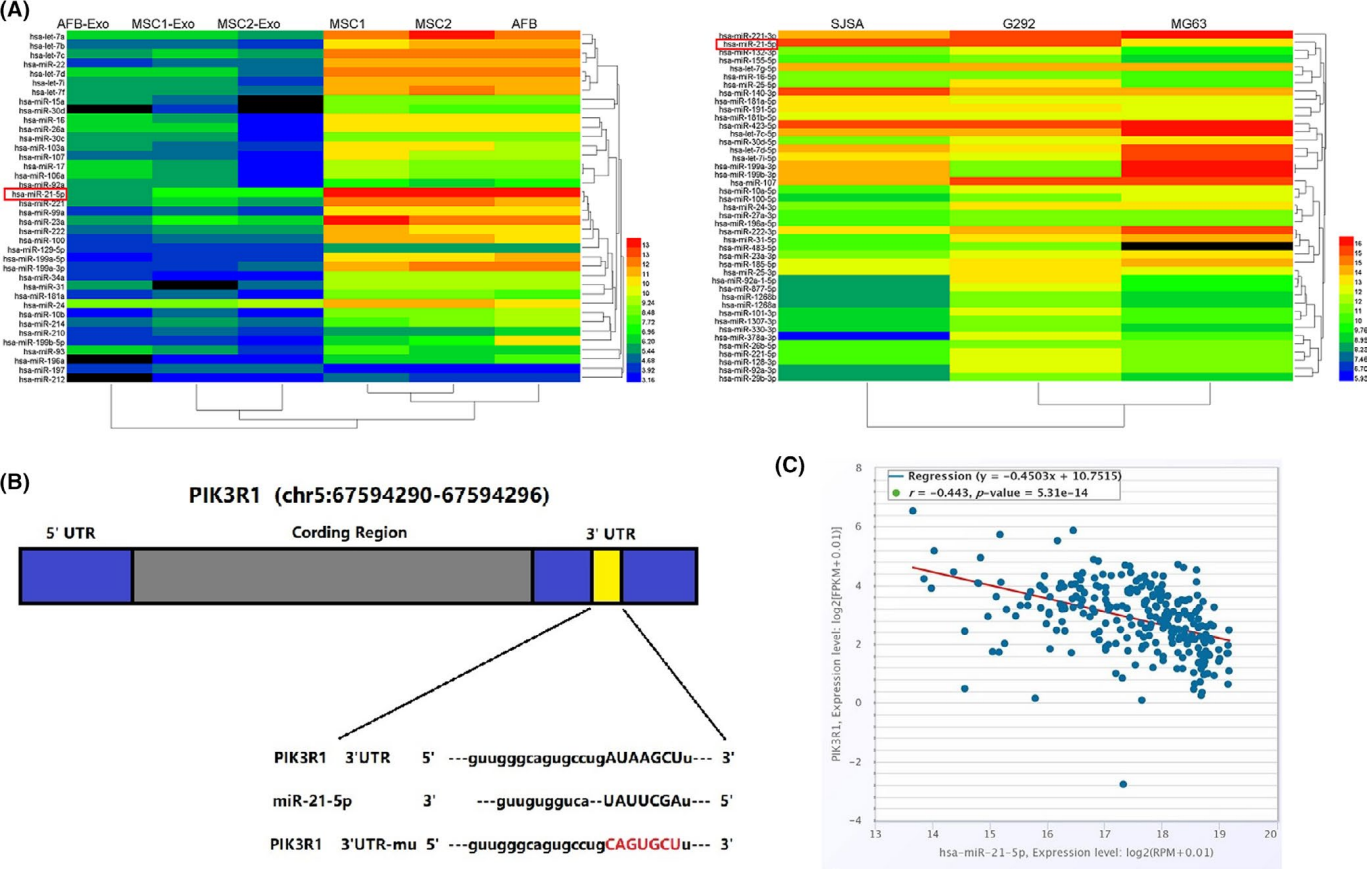
Expression of miRNA in human MSC and OS cells. (A) Microarray data were obtained from the Gene Expression Omnibus database (Accession No. GSE58027, GSE89930). Heat map shows significant upexpression of miR‐21‐5p in MSCs, MSC‐Exo and OS. (Blue represents low expression, red indicates high expression, and black indicates no significant change in gene expression. The colour change from blue to red indicates up‐regulation, and the colour change from red to blue indicates down‐regulation). (B) Schematic diagram of the miR‐21‐5p binding site on PIK3R1, bioinformatics analysis using miranda, targetscan, encori and pictar indicated that the 3'UTR of PIK3R1 mRNA contains a complementary site for the seed region of miR‐21‐5p. (C) The pan‐cancer analysis found that there was highly negative correlation between miR‐21‐5p and PIK3R1 in sarcoma

After co‐culture of MSC‐derived exosomes with OS cell, the proliferation and invasion of OS cells increased. We predicted that MSC‐derived exosomes, as biological vehicle, play a vital role in interaction between MSCs and OS. To further study the different expressions of miRNA (miR) in MSC‐derived exosomes, the miRNA profiling by array is available in the Gene Expression Omnibus (GEO) database (login No. GSE58027, GEO, (http://www.ncbi.nlm.nih.gov/geo/). Compared with adult fibroblasts cells (AFB), miR‐21‐5p exhibited highly upregulated expression in MSC‐derived exosomes and MSCs (Figure 4A left). From Gene Expression Omnibus (GEO) database (login No. GSE89930), we also found miR‐21‐5p highly expression between OS cells (Figure 4A right). miR‐21‐5p plays the role of oncogene in human cancers,^25^ according to the prediction of miranda, targetscan, encori and pictar, and we found there was a miR‐21‐5p binding site in the 3'noncoding region of PIK3R1 mRNA (Figure 4B). The pan‐cancer analysis from encori database found that there was highly negative correlation between miR‐21‐5p and PIK3R1 in sarcoma (Person *r* = −0.443, *P* = 5.31E‐14; *y* = −0.4503X + 10.7515) (Figure 4C).

### 3.5 Down‐regulation of PIK3R1 mRNA expression in human sarcoma and high‐regulated expression of MIR‐21‐5p in MSC‐derived exosomes and OS cells

**FIGURE 5:**
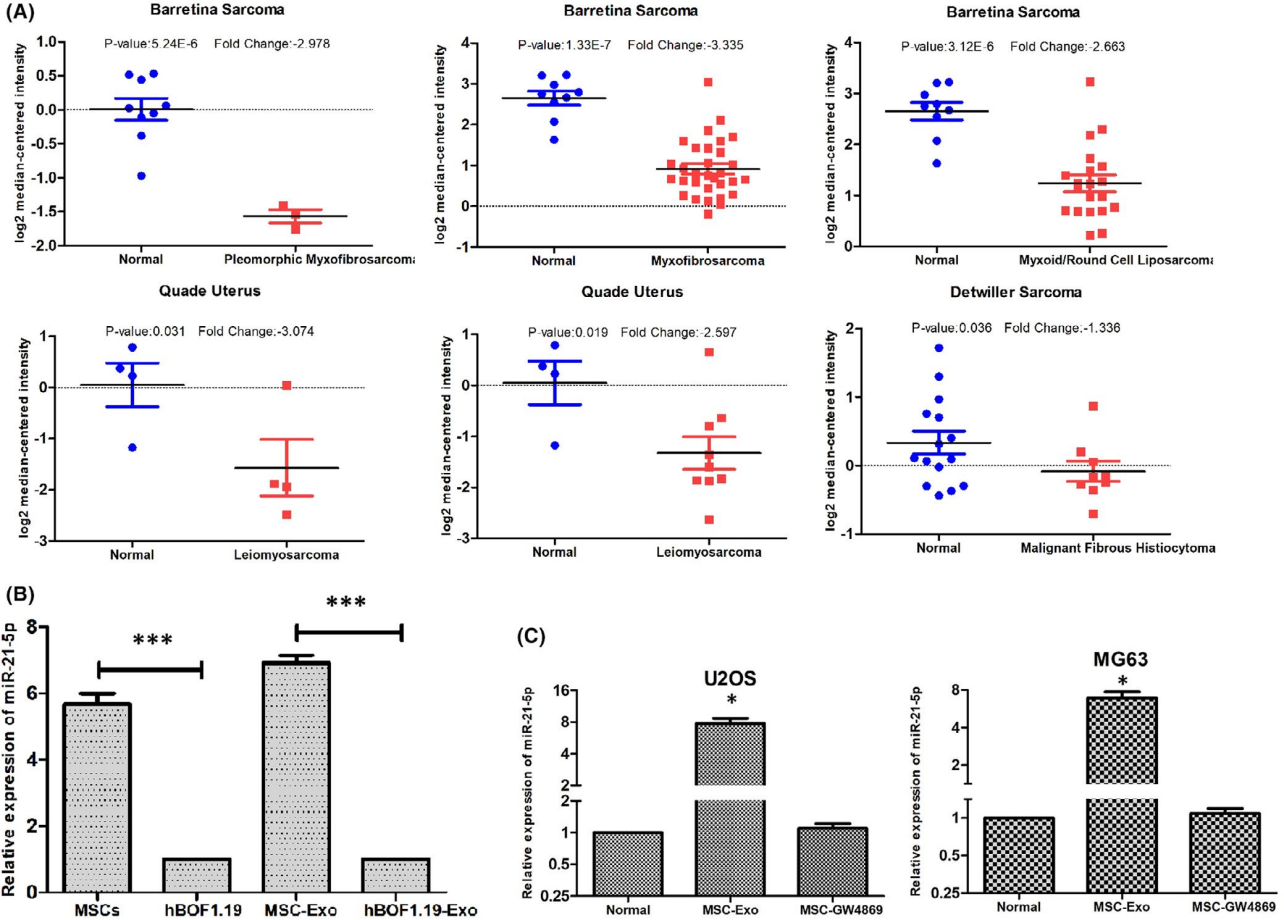
Expression of PIK3R1 is downregulated in human sarcomas, and the expression of miR‐21‐5p is upregulated in MSCs, MSC‐derived exosomes and OS cells. (A) Analysis of PIK3R1gene expressions in different subtypes of sarcomas using the Oncomine database. Box plot derived from gene expression data in the Oncomine database comparing the expressions of PIK3R1 between normal tissues and cancer tissues in different subtypes of sarcomas, pleomorphic myxofibrosarcoma, myxofibrosarcoma, myxoid/round cell liposarcoma, leiomyosarcoma and malignant fibrous histiocytoma. (B) The expression of miR‐21‐5p in MSCs, hFOB1.19 and their purified exosomes examined by RT‐qPCR. (C) The expression of miR‐21‐5p in U2OS and MG63 transfected with MSC‐derived exosomes and MGC‐GW8469 for 48 h examined by RT‐qPCR. Quantitative data from three independent experiments are shown as the mean ± SD (error bars). *p < 0.05, ***p < 0.001 (Student's *t* test)

We analysed the expression of PIK3R1 in human different sarcomas using Oncomine database. Cancer and normal samples from different patient datasets showed that PIK3R1 expression was significantly lower in pleomorphic myxofibrosarcoma, myxofibrosarcoma, myxoid/round cell liposarcoma, leiomyosarcoma and malignant fibrous histiocytoma (Figure 5A).

To determine whether MSC‐derived exosomes contributed to aberrant expression of miR‐21‐5p, we performed a qRT‐PCR for verification. The results indicated that the expression of MSCs‐ and MSC‐derived exosomes’ miR‐21‐5p was significantly higher than that of hFOB1.19 cell (*p* < 0.05) (Figure 5B). On the contrary, we use qRT‐PCR further detection of miR‐21‐5p expression in U2OS and MG63. Post‐co‐culture with MSC‐derived exosomes and MSC‐GW8469 for 48 h, the miR‐21‐5p expression was correspondingly changed in U2OS and MG63. Compared with the normal group (OS cells without treatment), the expression of miR‐21‐5p in U2OS and MG63 was significantly upregulated after co‐culture with MSC‐derived exosomes. Moreover, the expression of miR‐21‐5p in the MSC‐GW8469 group had no statistical difference compared with the expression in the normal group (Figure 5C). These results suggested that the expression of miR‐21‐5p in OS cells could be effectively regulated by MSC‐derived exosomes.

### 3.6 Exosomal MIR‐21‐5p derived from MSCs can accelerate OS cell proliferation and invasion

**FIGURE 6:**
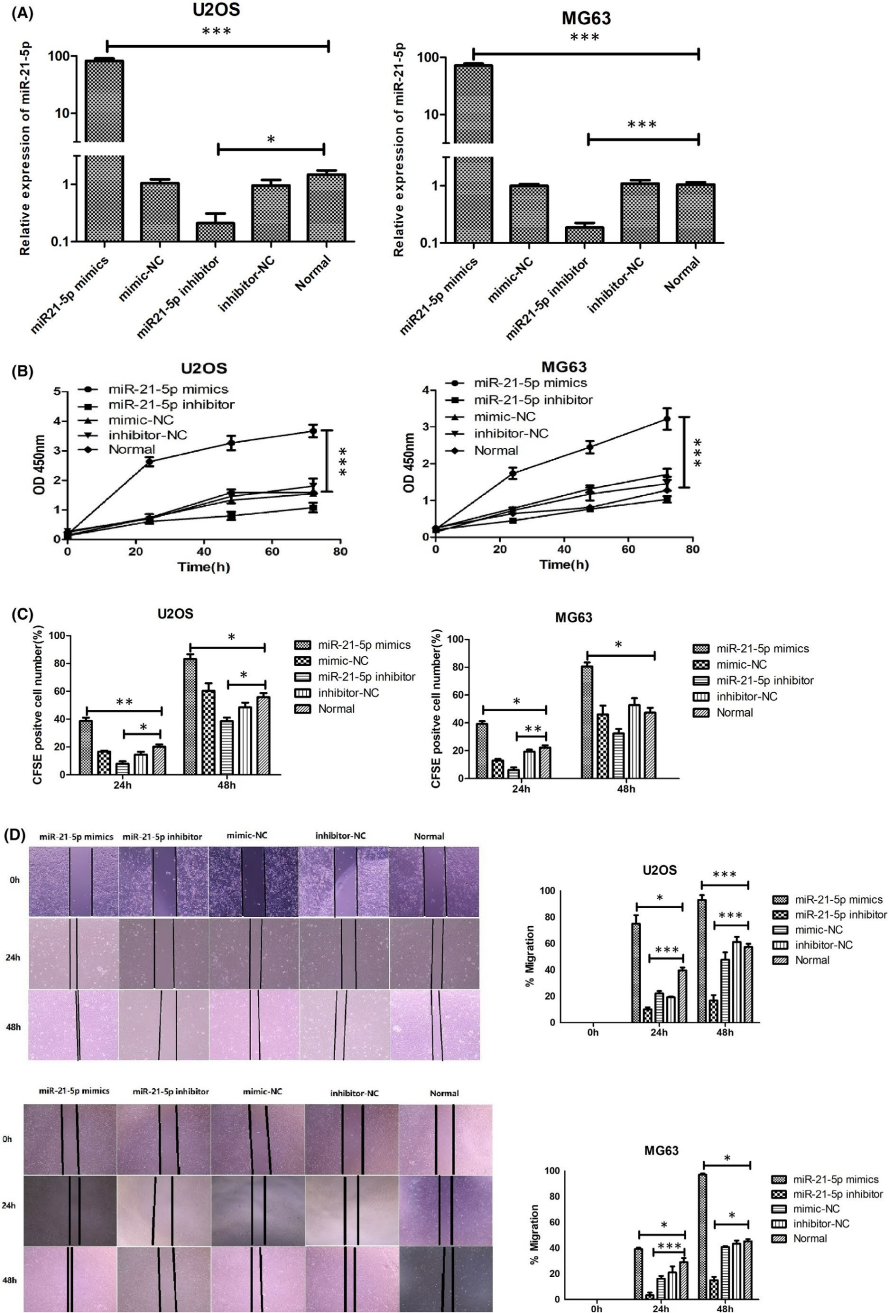
Exosomal miR‐21‐5p derived from MSCs promotes OS cells proliferation and invasion. (A) Expression of miR‐21‐5p in cultured U2OS and MG63 cells treated with different exosomes derived from different kinds of MSCs (MSCs treated with synthesized miR‐21‐5p mimic, miR‐21‐5p inhibitor, mimic‐NC, inhibitor‐NC and without any treatment). (B) U2OS and MG63 cells proliferation following miR‐21‐5p mimic treatment and miR‐21‐5p inhibitor examined by CCK‐8 assay. (C) CFSE fluorescence labelling system further evaluates U2OS and MG63 cells proliferation in different group. (D) The effect of exosomal miR‐21‐5p on the invasiveness of U2OS and MG63 was further evaluated by scratch‐healing experiment. Data were analysed via Student's t test. *p < 0.05, ***p < 0.001. miR, microRNA; NC, negative control

To verify the prediction of bioinformatics analysis that MSCs might enhance the proliferation and invasion of OS cells by transferring exosomal miR‐21‐5p into OS cells, MSCs were treated with miR‐21‐5p inhibitor or miR‐21‐5p mimics for 24 h, then purified the MSC‐derived exosomes, which treated U2OS and MG63 for 24 h. The levels of miR‐21‐5p were quantified using qRT‐PCR. The results showed that the level of miR‐21‐5p in U2OS and MG63 cultured with MSC‐derived exosomes (MSC treated with miR‐21‐5p inhibitor) was much lower compared with that in the normal group (*p *< 0.05). The level of miR‐21‐5p in U2OS and MG63 internalized with MSC‐derived exosomes (MSCs treated with miR‐21‐5p mimic) was much higher compared with that in the normal group (*p* < 0.001). In addition, we did not find significant changed in miR‐21‐5p expression in U2OS and MG63 cells between the negative control groups and normal groups (*p *> 0.05; Figure 6A). In the following experiments, we explored whether MSCs enhance the proliferation and invasion of U2OS and MG63 by transferring exosomal miR‐21‐5p. The proliferative abilities of U2OS and MG63 were evaluated by CCK8 assays. Compared with the normal groups, U2OS and MG63 internalized with the MSC‐derived exosomes (MSCs treated with miR‐21‐5p mimic) remarkably increased (*p *< 0.05). However, compared with the normal groups, OS cells internalized with MSC‐derived exosomes (MSCs treated with miR‐21‐5p inhibitor) have no significantly increase (*p *≥ 0.05, Figure 6B). The results were further validated by CFSE fluorescence labelling system, and the results revealed that OS cells internalized with the MSC‐derived exosomes (MSCs treated with miR‐21‐5p mimic) proliferative abilities remarkably increased, compared with the normal groups (*p* < 0.05), while U2OS and MG63 cells proliferative abilities remarkably decreased when treated with the MSC‐derived exosomes (MSCs with miR‐21‐5p inhibitor) (Figure 6C). Scratch wound‐healing assay showed that compared with that of the normal groups, the rates of wound healing in U2OS and MG63 cells were statistically accelerated in miR‐21‐5p mimic group (*p* < 0.05). Compared with that of the normal groups, the rates of wound healing were significantly attenuated in the miR‐21‐5p inhibitor group (*p* < 0.05, Figure 6D). The above findings suggested that up‐regulation of exosomal miR‐21‐5p expression in MSCs could significantly enhance the proliferation and invasion abilities of OS cells internalizing MSC‐derived exosomes, while down‐regulation of exosomal miR‐21‐5p in MSCs could attenuate these abilities of OS.

### 3.7 Exosomal MIR‐21‐5p derived from MSCs promote tumour growth in vivo

**FIGURE 7:**
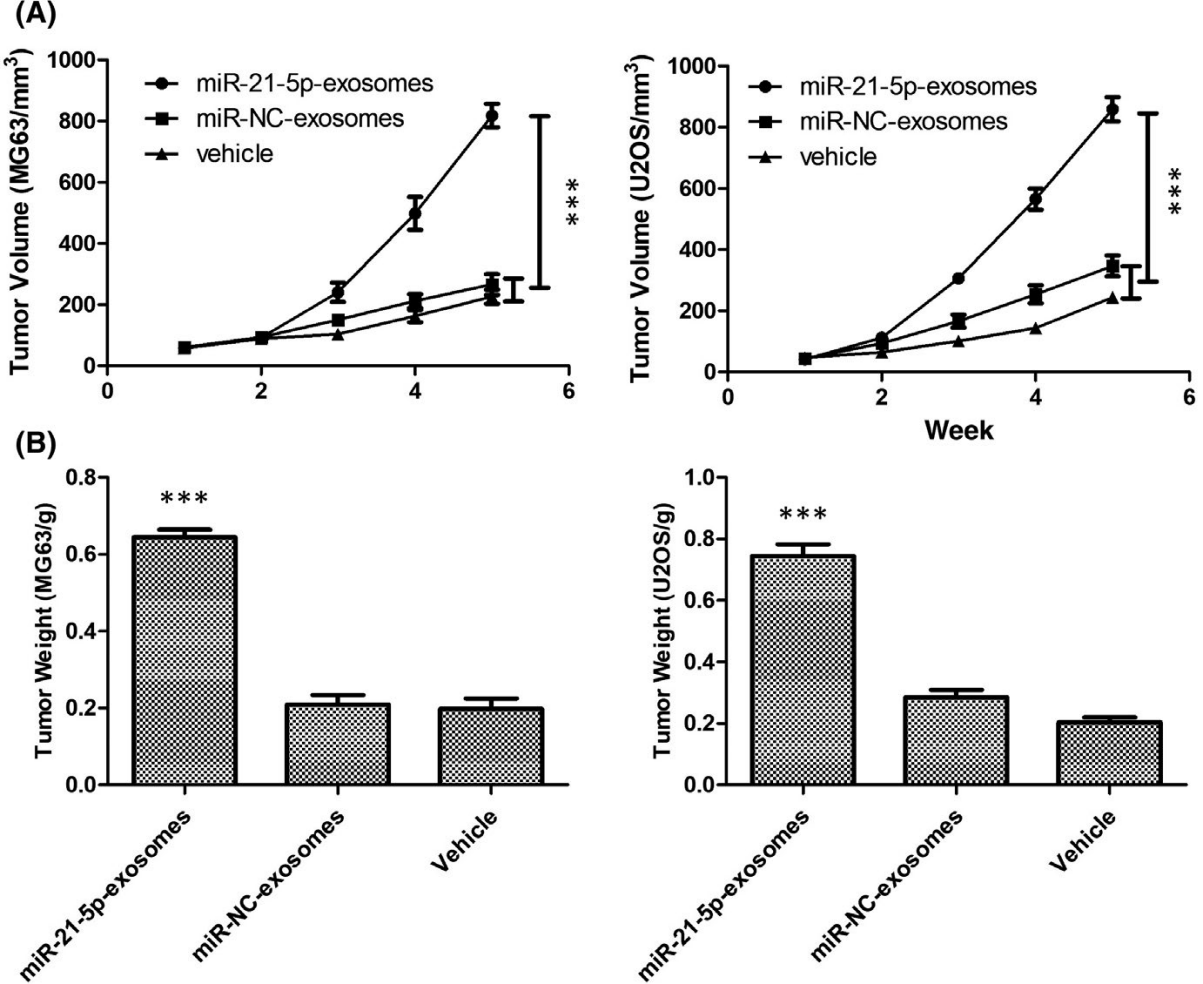
In vivo effect of miR‐21‐5p‐exosomes on tumour growth. (A) After tumour growth, tumour size was measured weekly by calliper. (B) Measure of tumour weight excised at sacrifice, treated with miR‐21‐5p‐exosomes or miR‐NC‐exosomes from MSCs or vehicle alone. Results are expressed as mean ± SD of six tumours for each experimental condition. ***p < 0.001 versus vehicle alone. Data were analysed via Student's *t* test

To study the effect of exosomal miR‐21‐5p derived from MSCs on OS growth in vivo, all mice were injected subcutaneously with MG63 and U2OS cells. After 1 week of OS growth, miR‐21‐5p‐exosomes, miR‐NC‐exosomes and the same volume of vehicle were weekly administered via intra‐tumour injection into mice. In the second week, there was no difference in tumour volume and weight in each group (*p *> 0.05, Figure 7A). In the 4th week, intra‐tumour injection of miR‐21‐5p‐exosomes significantly increased the tumour volume and weight compared with miR‐NC‐exosomes and vehicle‐treated control (*p *< 0.05, Figure 7A). At the sacrifice stage, the tumour volume and weight were significantly greater in mice treated with miR‐21‐5p‐exosomes than in controls injected with miR‐NC‐exosomes or with the vehicle alone (*p *< 0.05, Figure 7A,B).

### 3.8 Exosomal MIR‐21‐5p directly targets PIK3R1 to activate PI3K/AKT/MTOR signalling pathway

**FIGURE 8:**
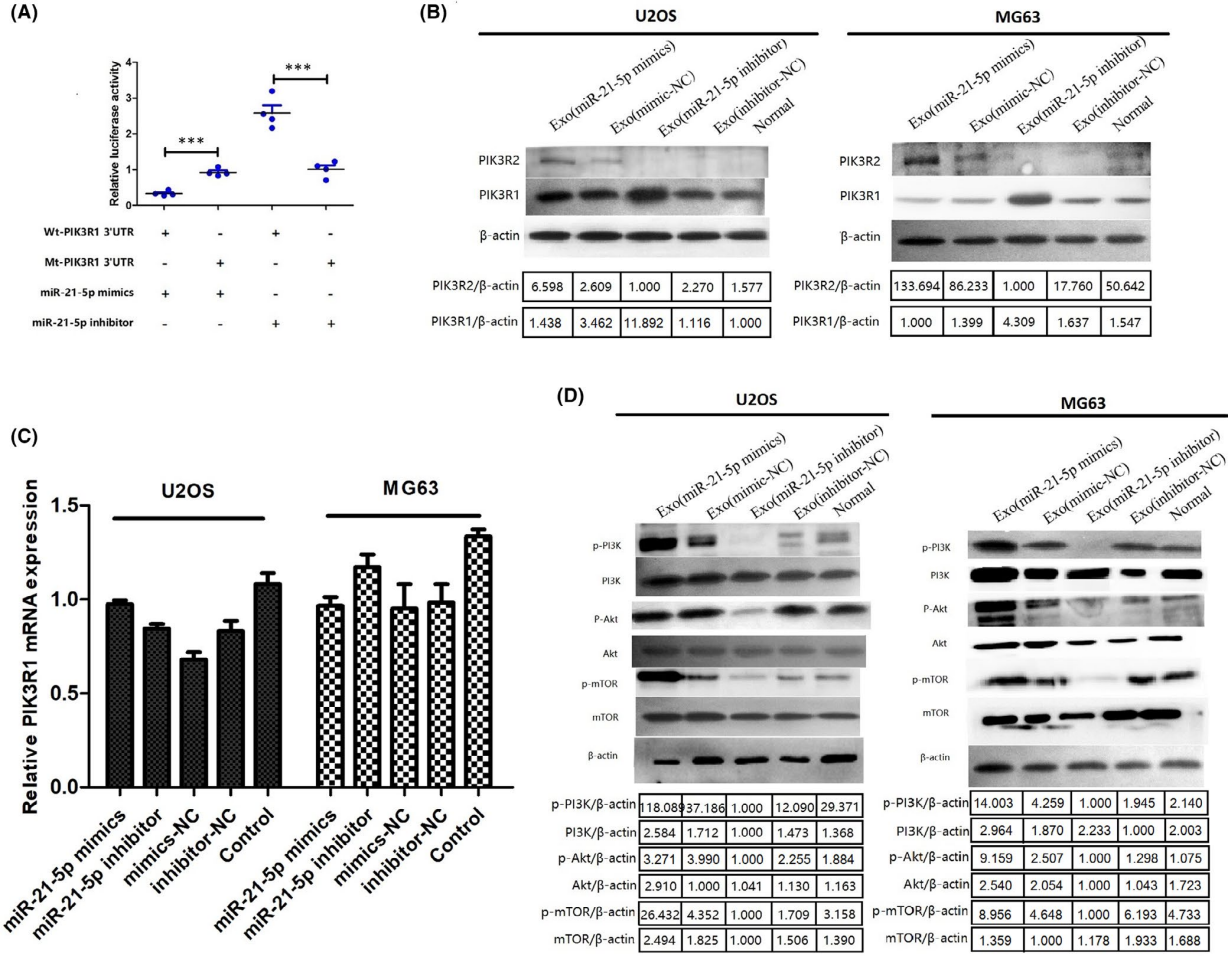
Exosomal miR‐21‐5p negatively regulated PIK3R1 and then activated the PI3K/AKT/mTOR signal pathway. (A) Luciferase reporter assays were conducted for validating the interaction between miR‐21‐5p and the 3’UTR of PIK3R1 (*n* = 4 independent experiments, one‐way ANOVA). (B)The Western blot assay showed the protein expression level of PIK3R1 was reduced by miR‐21‐5p overexpression of MSC‐derived exosomes inU2OS and MG63 cells. β‐actin was used as an internal control. (C) RT‐PCR showed the expression level of PIK3R1 has no statistical difference between miR‐21‐5p inhibitor groups and miR‐21‐5p mimics groups. (D) Western blot assay was implemented to measure the expression levels of p‐ or not PI3K, Akt and mTOR in U2OS and MG63 after treatment vs normal group. All experiments were implemented in triplicate, *p < 0.05, ***p < 0.001. mt, mutant; NC, negative control; p‐, phosphorylated; wt, wild type

To determine the targeting relationship between miR‐21‐5p and PIK3R1 3'UTR, we performed dual‐luciferase reporter assay system and found PIK3R1 was a direct target of miR‐21‐5p (Figure 8A). In addition, Western blot analysis found that miR‐21‐5p inhibition of MSC‐derived exosomes enhanced PIK3R1 protein levels, and miR‐21‐5p overexpression of MSC‐derived exosomes induced the opposite effects in U2OS and MG63 cells. However, there were no significant differences in PIK3R2 expression between groups (Figure 8B). The results of RT‐PCR showed that the expression level of PIK3R1 has no statistical difference between miR‐21‐5p inhibitor group and miR‐21‐5p mimics group (Figure 8C). This suggested that miR‐21‐5p inhibits the expression of PIK3R1 at post‐transcriptional level.

To gain further mechanistic insights into the roles of miR‐21‐5p/PIK3R1 axis, we hypothesized that exosomal miR‐21‐5p derived from MSCs activates the PI3K/Akt/mTOR signalling pathway in OS, leading to OS proliferation and invasion, because the PI3K regulatory subunit p85α/PIK3R1 can exert a tumour‐suppressor effect by negatively regulating the PI3K‐Akt signalling pathway.^28^ Using the Western blot analysis, the expression levels of p‐Akt, p‐mTOR and p‐PI3K in MSC‐derived exosomes‐treated OS cells (MSCs treated with miR‐21‐5p inhibitor) were prominently decreased, while the expression of PIK3R1was significantly increased. After treatment with the MSC‐derived exosomes (MSCs treated with miR‐21‐5p mimics), the expression levels of p‐Akt, p‐mTOR and p‐PI3K in OS cells all showed an increasing trend, whereas the expression of PIK3R1 sharply decreased (*p *< 0.05). However, the expressions of Akt, mTOR and PI3K were relatively stable in OS cells (Figure 8D).

**FIGURE 9:**
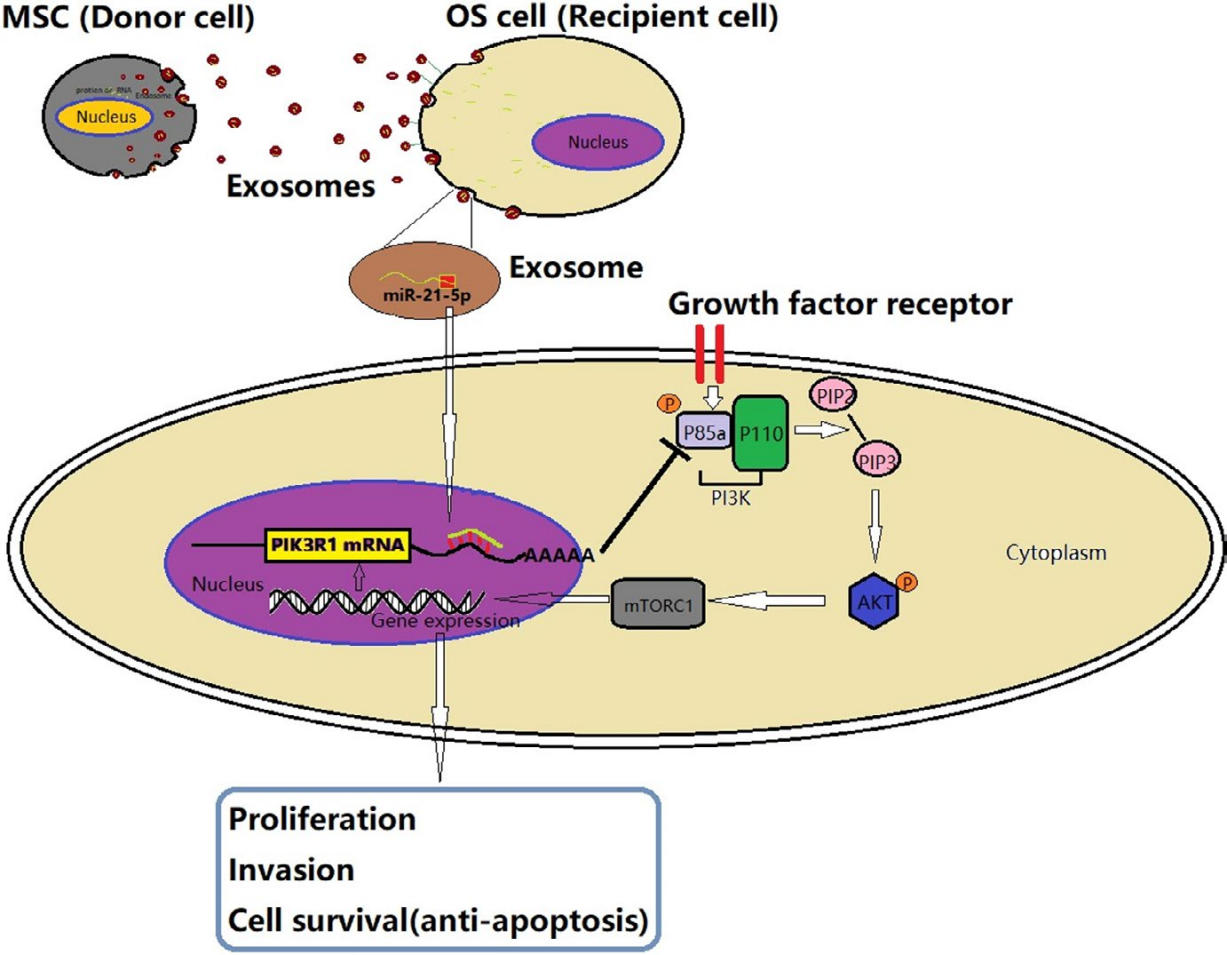
Schematic of the action of exosomal miR‐21‐5p derived from human bone marrow MSCs regulating OS cells progression. Human bone marrow MSCs transmit miR‐21‐5p to OS cells through exosomes, exosomal miR‐21‐5p target PIK3R1 gene of OS cells, then activating PI3K/Akt/mTOR signal pathway and leading to OS proliferation, invasion and anti‐apoptosis

In summary, the results indicated that bone marrow MSCs could produce amounts of exosomes, which may deliver miR‐21‐5p into OS cells and thus promote OS proliferation and invasion. The increased miR‐21‐5p in OS cells specifically targeted the 3′UTR region of PIK3R1 mRNA, leading to the decrease in p85α protein level and the activation of corresponding PI3K/Akt/mTOR signalling pathway. A detailed summary of the results is showed in Figure 9.

## 4 DISCUSSION

Recently, more and more studies have shown that exosomes play a vital role in information transfer between cells^16^, ^45^ and released by many types of cells.^46^ Exosomes are the main type of small extracellular vesicles (sEVs), and they contain a large number of bioactive substances that can be transferred from donor cells to recipient cells, which trigger the transformation of signalling pathway and cell phenotypic of recipient cells.^47^ MSCs have been used in previous studies in relation to cancer ailment because the properties of MSCs exist in tumour‐associated microenvirionment and have a homing ability that allows them to migrate to sites of cancer.^48^ Bone marrow MSCs are a kind of pluripotent stem cells, which can secrete a large number of exosomes and act as paracrine mediators in tumour‐related microenvironment. However, the role of MSC‐derived exons in the pathogenesis and progression of cancer cells, especially OS, has not been fully clarified.

Bone marrow MSCs that can secrete a large number of exosomes are a type of pluripotent stem cells that act as paracrine mediators in tumour‐related microenvironment. However, the role of MSC‐derived exosomes in the pathogenesis and progression of cancer cells, especially OS, has not yet been fully elucidated. Interestingly, there are contradictory reports that MSCs promote cancer growth. MSC‐derived exosomes contain tumour supportive miRNAs, tumour supportive factors, bioactive lipids, lactic acid and glutamic acid, which support breast cancer cell proliferation and metastasis,^49^ whereas MSC‐derived exosomes induce tumour‐associated microenvironment and endow gastric cancer cells with stemness by activating Akt signalling pathway.^50^ Moreover, study has found that bone marrow MSCs and multiple myeloma cells mutually communicate through exosomes, and MSC‐derived exosomes enhance multiple myeloma cell progression, metastasis and drug resistance.^51^ However, MSC‐derived exosomes may have completely different effects on the proliferation of different types of tumour cells. Some reports showed that MSC‐derived exosomes inhibit tumour initiation and progression.^42^, ^52^, ^53^ Therefore, it is very important to study the exact mechanism of MSC‐derived exosomes on OS proliferation and migration. Our study demonstrated the invasion and migration effects of MSC‐derived exosomes in OS cells and examined which signalling pathway is influenced by MSC‐derived exosomes so as to understand the potential molecular mechanism on OS.

The extraction of exosomes was the main difficulty of this study. The main methods of exosome separation and extraction are gradient ultracentrifugation, kit extraction, ultrafiltration, density gradient centrifugation, polymeric precipitation and immunomagnetic beads.^54^ In this experiment, we purified MSC‐derived exosomes by gradient ultracentrifugation, which has the advantage of high purity. However, this method is complicated, costly and time‐consuming and it requires the laboratory to have an ultra‐speed centrifuge and its corresponding centrifugal rotor. We found that after OS cells were co‐cultured with MSCs, MSCs induced the proliferation and invasion of OS cells through MSC‐exosome delivery. The results showed that MSC‐derived exosomes could promote the proliferation and invasion of OS cells, and the expression of Bcl‐2 was significantly increased, and Bax was sharply decreased in MSC‐derived exosomes group. Studies have shown that Bcl‐2 inhibits cell apoptosis, while Bax can promote cell apoptosis. The imbalance of the ratio of Bcl‐2 and Bax is closely related to cell apoptosis or inhibition.^55^ Bax can induce mitochondrial membrane changes and activate downstream caspase‐3 cascade reaction when cells are exposed to apoptosis signal and then induce apoptosis.^56^

To further study the mechanism of MSC‐derived exosomes on OS cell proliferation and invasion, we found that in OS‐associated microenvironment, MSC‐derived exosomes express high level of miR‐21‐5p, which can be regarded as oncogene in cancer.^25^, ^57^ Moreover, study have found that miR‐21‐5p is over‐expressed in various types of tumours, and it plays an important role in tumorigenesis and tumour progression; high miR‐21‐5p expression is related to poor prognosis and short survival.^25^ Does MSCs in OS‐associated microenvironment involved in OS proliferation and invasion by mediating exosomal miR‐21‐5p? At present, it has not been studied. We found transfection of miR‐21‐5p mimics or miR‐21‐5p inhibitor into MSCs could significantly up‐ or downregulate the expression of miR‐21‐5p in OS cells and can significantly promote or attenuate the proliferation and invasion of OS. High expression of miR‐21‐5p in primary OS can be used as an indicator of diagnosis and prognosis of OS.^58^ Importantly, we firstly found MSC‐derived exosomal miR‐21‐5p promotes OS proliferation and invasion by targeting PIK3R1 and activity of cancer‐related PI3K/Akt/mTOR signalling pathway. PIK3R1 was downregulated and PI3K/Akt/mTOR signalling pathway was enhanced in OS cells following treatment of MSC‐derived exosomes containing miR‐21‐5p mimics. In addition, PIK3R1 was upregulated and PI3K/Akt/mTOR signalling pathway was inhibited when OS cells treated with MSC‐derived exosomes containing miR‐21‐5p inhibitor.

This study is the first to investigate the relationship between MSC‐exosomal miR‐21‐5p and targeting gene PIK3R1 in OS. Our study has revealed that PIK3R1 is the only down‐expressed member of PI3K in OS. PI3K is a key molecule in the PI3K/Akt/mTOR signalling pathway, which is involved in the regulation of a variety of transcription factors in cancer.^59^ PI3K is a heterodimer composed of a regulatory subunit (p85) and a catalytic subunit (P110). The regulatory subunit (p85) contains SH2 and SH3 domains and interacts with target proteins containing corresponding binding sites. According to the different regulatory subunits, class I PI3Ks are divided into three categories, with different structures and functions.^60^ PIK3R1 gene encode one of p85‐type subunits: p85α.^30^ PIK3R1/p85α is abundant isoform in tumour‐free tissues, but its expression is reduced in tumour cells.^31^, ^61^ Therefore, PIK3R1/p85α, as a tumour suppressor, can maintain the stability of p110a of PI3K. Loss of p85α leads to aberrant activation of the downstream PI3K pathway.^62^, ^63^, ^64^, ^65^ Therefore, the effect of down‐regulation of p85α may be due to the loss of the inhibitory effect of p85α on the activity of p110 and PI3K pathway. In spite of the extensive knowledge on class I PI3K, until recently, the function and mechanism of PIK3R1 in the proliferation and invasion of malignant tumours are not clear.

In conclusion, this report offers a comprehensive analysis of exosome that sheds light on the cell communication in OS‐associated microenvironment. The results of our study demonstrate that there were specific conserved sites between miR‐21‐5p and 3’‐UTR of PIK3R1, and PIK3R1 down‐regulation by MSC‐derived exosomes containing abundant miR‐21‐5p exerts tumour promoter properties in OS. All these results support the notion that exosomal miR‐21‐5p derived from MSCs exerts tumour promoter properties in OS by down‐regulating PIK3R1 so as to activate the PI3K/Akt/mTOR signalling pathway.

## CONFLICT OF INTERESTS

The authors declare that they have no competing interests.

## AUTHOR CONTRIBUTION

**Jin Qi:** Conceptualization; Data curation; Formal analysis; Funding acquisition; Investigation; Methodology; Project administration; Resources; Software; Supervision; Validation; Visualization; Writing‐original draft; Writing‐review & editing. **Ruihao Zhang:** Data curation (equal); Investigation (equal); Methodology (equal); Software (equal). **Yapeng Wang:** Formal analysis (equal); Methodology (equal).

## CONSENT FOR PUBLICATION

Not applicable.

## Data Availability

All data generated or analysed during the study are included in this published article. GEO data were downloaded from the GEO datasets (https://www.ncbi.nlm.nih.gov/gds/).
